# Antimicrobial Susceptibility Patterns of *Staphylococcus* spp. Isolates from Mastitic Cases in Romanian Buffaloes from Western Romania

**DOI:** 10.3390/antibiotics14060537

**Published:** 2025-05-23

**Authors:** János Degi, Viorel Herman, Ionica Iancu, Corina Badea, Cristian Zaha, Petru Eugen Mergheș, Vlad Iorgoni, Bogdan-Alexandru Florea, Romeo Teodor Cristina, Diana Maria Degi

**Affiliations:** 1 Department of Infectious Diseases and Preventive Medicine, University of Life Science “King Michael I”, 300645 Timisoara, Romania; viorelherman@usvt.ro (V.H.); ionica.iancu@usvt.ro (I.I.); corina.badea@usvt.ro (C.B.); vlad.iorgoni@usvt.ro (V.I.); 2 Department of Surgery, University of Life Science “King Michael I”, 300645 Timisoara, Romania; cristian.zaha@usvt.ro; 3 Department I, Faculty of Animal Husbandry and Biotechnology, University of Life Science “King Michael I”, 300645 Timisoara, Romania; petrumerghes@usvt.ro; 4 Department of Internal Medicine, University of Life Science “King Michael I”, 300645 Timisoara, Romania; bogdan.alexandru-florea@usvt.ro; 5 Department of Pharmacy and Pharmacology, University of Life Science “King Michael I”, 300645 Timisoara, Romania; romeocristina@usvt.ro; 6 Department of Toxicology and Toxicoses, Plant Biology and Medicinal Plants, University of Life Science “King Michael I”, 300645 Timisoara, Romania; diana.maria-degi.fbira@usvt.ro

**Keywords:** milk, Romanian buffalo, mastitis, *Staphylococcus aureus*, causative agent

## Abstract

Mastitis is defined as mammary gland inflammation and is one of the most common and economically significant diseases affecting dairy cows. Bacteria are the most frequently reported agents responsible for mastitis, while other pathogens are often overlooked due to insufficient routine investigation. Incomplete diagnoses can result in inappropriate antimicrobial treatments, treatment failures, antimicrobial resistance, the spread of pathogens, and the recurrence of mastitis. **Background/Objectives**: This study aimed to investigate the presence of *Staphylococcus* spp. associated with Romanian buffalo mastitis on dairy farms in Western Romania via a bacteriological analysis of mastitis milk and determine antimicrobial susceptibility profiles. **Methods**: Bacterial culture was performed according to the guidelines described by the National Mastitis Council. Vitek 2 Compact systems (Bio Mérieux, France), with the GP ID cards, were used to confirm the species of the isolates. Antibiotic susceptibility testing was conducted by utilizing Vitek^®^ 2 preset antimicrobial card AST-GP79 Gram-positive Livestock WW. **Results**: Of all the milk samples (*n* = 115) analyzed, 83 were positive for *Staphylococcus* spp. (72.17%) and were evaluated for their antimicrobial susceptibility profiles. The most common microorganism found was *S. aureus* (*n* = 46; 55.42%), followed by *S. hyicus* (*n* = 28; 33.73%) and *S. schleiferi* (*n* = 9; 10.84%). These pathogens demonstrated significant resistance to the tetracycline, neomycin, benzylpenicillin, and erythromycin. **Conclusions**: Current control measures for mastitis caused by *S. aureus* are ineffective. A better understanding of the virulence factors in Romanian buffalo-adapted strains of *S. aureus*, their pathogenesis, and host immunological responses is essential for developing effective and sustainable non-antibiotic control tools such as vaccines, prophylactic therapies, and other innovative approaches.

## 1. Introduction

Buffaloes represent a species of significant economic interest due to the products obtained from them. Thus, buffalo milk, which accounts for 12% of global milk production, ranks second after cow’s milk [1,2]. It has a high nutritional value and is used to make mozzarella cheese and other products. Similarly, buffalo meat is of exceptional quality and is noted for its low fat and cholesterol content [3]. Raising buffalo is a traditional activity with a long history in our country, and interest in buffalo farming remains especially strong in Central and Western Romania, where buffalo are raised on farms and homesteads [4,5]. The Banat region is known for the many buffalo growing there [6]. Maintaining proper health status is one of the essential conditions for raising any species and achieving high production levels. Infectious diseases in buffalo are particularly interesting, as they can impact their health and productivity [7].

Economic development, poverty alleviation, and rural livelihood are all significantly influenced by the buffalo industry. The population’s swiftly increasing demand for animal protein can also be partially met by it [8,9]. The industry is confronted with new challenges, despite the significant contributions of buffaloes to agriculture and human well-being [10].

Mastitis is recognized as one of the most prevalent and economically significant diseases impacting lactating dairy herds globally in the field of veterinary medicine [11]. The increasing demand for buffalo milk and its dairy products has led to efforts aimed at enhancing buffalo milk yield, which has consequently contributed to a rise in the incidence of mastitis among buffaloes [8]. Nonetheless, there is a deficiency of pertinent research regarding buffalo mastitis. Mastitis typically leads to inflammation in one or more quarters of the mammary glands, impacting not just an individual animal but potentially the entire herd or several members within it [8,12]. Efficient, precise, and cost-effective methods for diagnosing mastitis are crucial in minimizing the losses associated with this condition.

Mastitis typically arises from bacterial infections. Over 130 bacterial species have been linked to bovine mastitis [13,14,15]. Nonetheless, several bacterial species, including *S. aureus*, *Streptococcus uberis*, *Streptococcus dysgalactiae*, and *Escherichia coli*, account for around 80% of mastitis cases [8,15]. Additional coagulase-positive staphylococci (CPS) and coagulase-negative staphylococci (CNS) have been commonly linked to mastitis [16,17,18,19,20,21]. *Staphylococcus aureus* can enter milk through direct excretion from udders affected by clinical or subclinical staphylococcal mastitis or through environmental contamination while handling raw milk [13]. When the udder is infected, *S. aureus* may be present in the milk in varying amounts, reaching up to 10^8^ CFU/mL [14,15]. Antibiotic-resistant bacteria have emerged as a significant global public health concern, affecting both human and veterinary medicine [16,17].

The indiscriminate use of antibiotics in treating animal and human diseases and milk preservatives have led to the development of microorganisms resistant to antibiotics, rendering antibiotic treatment ineffective. *S. aureus* has frequently been reported to exhibit multiple patterns of antimicrobial resistance.

The strategy for preventing and addressing cow mastitis cases generally includes using antimicrobials [22,23]. It is important to note that numerous bacterial isolates have demonstrated resistance to three or more classes of antimicrobials, underscoring the issue of multidrug resistance (MDR) [8,15]. In Western Romania, small buffalo farms that depend on family labor are essential for milk production for processing industries. A significant obstacle in these farms is the absence of technical support, which can result in shortcomings in animal management that contribute to intramammary infections leading to buffalo mastitis, as treatment is frequently carried out based on empirical technical advice [24]. The implementation of these practices could elevate the risk of antimicrobial resistance, leading to the dissemination of resistant bacterial strains within the community [18].

Observing the susceptibility patterns of clinical isolates is an essential component of the One Health approach [25]. Several investigations indicate that susceptibility testing is beneficial for choosing the most effective agents to address mastitis infections. Nonetheless, the accuracy of susceptibility testing in vitro is constrained when it comes to predicting the curability of mastitis [26]. Nevertheless, the guidelines set forth by the European Commission for the careful use of antimicrobials in veterinary medicine advocate for conducting susceptibility testing prior to administering antimicrobials for mastitis treatment [27]. This approach aims to mitigate the dissemination of resistant bacteria by ensuring the selection of an appropriate antimicrobial is based on rational criteria [25]. Nonetheless, there is a notable scarcity of peer-reviewed publications addressing antimicrobial resistance in Romanian buffalo within Romania.

Thus, this study aimed to isolate, identify, and determine the antimicrobial susceptibility profiles of *Staphylococcus* spp. in clinical mastitis in the Romanian buffalo in the study area.

## 2. Results

### 2.1. Bacteriological Results

Out of the 115 milk samples studied, 83 tested positive for *Staphylococcus* spp. (72.17%; 95% CI, 63.98–80.36%; *p*-value < 0.05) and were assessed for their antimicrobial susceptibility profiles. Three different species of *Staphylococcus* were found. *Staphylococcus aureus* was the most frequently identified microbe (*n* = 46; 55.42%; 95% CI, 44.72–66.12%; *p*-value < 0.002), followed by *Staphylococcus hyicus* (*n* = 28; 33.73%; 95% CI, 23.54–43.92%; *p*-value < 0.95) and *Staphylococcus schleiferi* (*n* = 9; 10.84%; 95% CI, 4.06–17.62%; *p*-value < 0.002). Table 1 illustrates the distribution of identified *Staphylococcus* species, as determined by the Vitek 2 GP ID card.

In the present study, all isolates underwent slide coagulase testing for clumping factors. All S. aureus strains were coagulase-positive in the tube coagulase test, and they reacted positively to the clumping factor test accordingly. Regarding the *S. hyicus* strains isolated in our study, the results are as follows: in the tube coagulase test, all strains were positive; however, in the slide test, only 7 out of the 26 isolated strains were positive. Instead, all nine strains of *S. shleiferi* were clumping factor-positive and tube coagulase-negative.

In the case of the test for urease activity detection, the results indicated that all tested strains were urease-negative, confirming their classification as *S. schleiferi* and ruling out the possibility of misidentification.

### 2.2. Results of the Antimicrobial Susceptibility Test

All 83 isolates examined demonstrated satisfactory growth in the control well of the Vitek 2 automated susceptibility testing (AST) card. The results were automatically aligned with the criteria established by the Clinical and Laboratory Standards Institute (CLSI) for antimicrobial susceptibility test, as outlined in CLSI M100-S24 [26], and in accordance with the technical data sheet of the Vitek AST-GP79 card (Ref. 421825). According to the Laboratory Report, the interpretation for each sample is performed automatically.

The antimicrobial susceptibility profiles of the identified *Staphylococcus* spp., as assessed by the Vitek^®^ 2 system, are detailed in Table 2. The most significant prevalence of resistant *S. aureus* isolates was observed concerning tetracycline (37/46; 80.43%; *p*-value < 0.01), neomycin (33/46; 71.73%), and benzylpenicillin (29/46; 63.04%). For *S. hyicus*, the highest resistance rates were found for tetracycline (24/28; 85.71%; *p*-value < 0.01), neomycin (21/28; 75.0%), and erythromycin (20/28; 71.42%). Tetracycline, neomycin, and benzylpenicillin (7/9; 77.78%; *p*-value < 0.01) presented the highest resistance rates for *S. schleiferi*.

Upon analyzing the antimicrobial susceptibility patterns by class, the highest rate of antimicrobial resistance was observed in tetracyclines (68/83, 81.92%), macrolides (54/83, 65.06% for erythromycin; 47/83; 56.62% at tilmicosin and 37/83; 44.57% at tylosin of the isolates), and aminoglycosides (61/83, 73.49% at neomycin; 52/83, 62.65% at gentamicin; 46/83, 55.42% at kanamycin; 41/83, 49.39% at amikacin; and 33/83, 39.76% at streptomycin of the isolates). The fluoroquinolones and β-lactams class displayed a low resistance rate (10/83, 12.04%, respectively, 27/83, 32.53% at ceftiofur; 23/83, 27.71% at ceftiofur; 19/83, 22.89% at oxacillin; and 2/83, 2.40% at cefquinome of the isolates) (Table 2). In the case of the beta-lactam class, the exceptions are two antibiotics, namely benzylpenicillin (53/83, 63.85%) and ampicillin (38/83, 45.78%), where higher resistance values were observed.

Multidrug-resistant (MDR) *Staphylococcus* isolates were identified in 73.5% (61/83) of buffalo mastitis cases, with *Staphylococcus schleiferi* exhibiting the highest prevalence of MDR (7/9, 77.8%), followed by *S. hyicus* (21/28, 75.0%) and *S. aureus* (33/46, 71.8%). Among the *S. aureus* isolates exhibiting resistance to up to 8 antimicrobial classes, 4 isolates exhibited resistance to 3 classes, 1 isolate to 4 classes, 11 isolates to 5 classes, 2 isolates to 6 classes, 6 isolates to 7 classes, and 9 isolates demonstrated resistance to 8 antimicrobial classes, predominantly β-lactams, aminoglycosides, macrolides, tetracyclines, and sulfonamides. *S. hyicus* displayed a similar resistance pattern, encompassing resistance to 7 antimicrobial classes (1 isolate showed resistance to 3 antimicrobial classes, 3 isolates to 4 classes, 4 isolates to 5 classes, 4 isolates to 6 classes, and 9 isolates exhibited resistance to 7 antimicrobial classes). Despite the lower number of isolates, *S. schleiferi* demonstrated a strong capacity for multidrug resistance, resistant to up to 8 antimicrobial classes (3 showed resistance to 5 antimicrobial classes, 2 to 6 classes, 1 to 7 classes, and 1 isolate demonstrated resistance to 8 antimicrobial classes). These findings indicate a high degree of variability in resistance levels, with a substantial proportion of strains exhibiting extensively resistant phenotypes.

The widespread MDR observed in these isolates underscores the urgent need for enhanced antimicrobial stewardship programs to control the spread of resistance and optimize treatment strategies in veterinary medicine. Table 3 presents the resistance patterns of *Staphylococcus* spp. (*n* = 83) isolated from buffalo mastitis cases, displaying resistance to more than three antimicrobials.

## 3. Discussion

Our results indicated a wide variability in the antimicrobial susceptibilities of *Staphylococcus* spp. isolated from Romanian buffalo with clinical mastitis in Western Romania. These pathogens exhibited high resistance to tetracycline, neomycin, benzylpenicillin, and erythromycin. In comparison, the previous cattle mastitis study demonstrated that approximately half of the *S. aureus* isolates were susceptible to amoxicillin [28].

The results obtained in our study show that all isolated staphylococcal species showed resistance to tetracycline, neomycin, benzylpenicillin, and erythromycin. These antibiotics are often used to treat mastitis in cattle and buffaloes in Romania. In most clinical cases, commercial products containing these antibiotics are often used without a medical prescription or veterinary assistance.

This aligns with findings from mastitis cases involving *S. aureus* documented in Europe and the United States, where beta-lactamase producers were found in 35.1% of *S. aureus* isolates [25,29,30]. Conversely, a prior study on antimicrobial resistance in mastitis conducted across European dairy farms found that resistance of *S. aureus* to ceftiofur was observed in merely 1% of the isolates [28]. Amid the increasing occurrence of resistant bacteria, amoxicillin continues to rank as one of the four most frequently utilized antimicrobials in veterinary medicine for the treatment of *S. aureus* infections in Romania [31,32].

The significant prevalence of ceftiofur- and cefotaxime-resistant *S. aureus* isolates found in this study indicates that there could be a spread of extended-spectrum beta-lactamase-producing strains across the examined buffalo farms, underscoring a crucial knowledge gap that necessitates additional research. This observation may be attributed to the uncontrolled application of extended-spectrum cephalosporins to farming husbandry in Western Romania, primarily owing to the preferential use of these antimicrobials to treat buffalo and bovine mastitis [24,33].

Given the considerable health risk associated with methicillin-resistant *S. aureus* (MRSA) in Europe and worldwide, it is advisable to monitor the prevalence of *S. aureus* infections in production animals [34,35]. To date, a previous study on dairy cattle has reported only three cases of MRSA in the Romania and Banat regions [24,36,37]. Regrettably, there is currently no program in place to monitor the prevalence of staphylococcal infections in animals within Romania.

Saeed et al. [38] indicated that infections from *S. aureus* constitute the predominant mastitis issue in dairy cattle due to the significantly low antibiotic cure rate during lactation. In numerous instances, the infections become chronic, necessitating the periodic culling of the sick animal. Mastitis induced by this disease can only be effectively managed through the prevention of new infections and the culling of sick animals. Similarly to other infectious agents, it is transmitted by milking-machine apparatus, the hands of milking staff, and washcloths [39].

Despite the susceptibility of *S. aureus* strains to various antibiotics in vitro, farmers frequently express concerns that the cure rates observed under in vivo conditions fall short of expectations. This observation is potentially supported by evidence indicating that *S. aureus* is capable of surviving under neutrophil activity [40], leading to fibrosis in the udder and the invasion of mammary epithelial cells [9]. Nonetheless, the main factor contributing to the low cure rate is its capacity to form microabscesses that hinder antibiotics from accessing the pathogen. Research findings indicate that the production losses resulting from staphylococcus mastitis tend to be long-term. The pathogen leads to irreversible harm to the udder’s secretory tissue, which is then replaced by non-secretory tissue, resulting in a reduction in the cow’s milk production capacity [41].

Cobirka et al. [42] indicated that while *S. aureus* may be transmitted from one cow to another, it can also persist in the dairy barn environment between milkings. Heifers serve as established reservoirs for this pathogen. Between 12 and 15 percent of first-lactation cows were found to be infected with *S. aureus* [43]. A significant number went unnoticed and became infected during lactation, acting as reservoirs for transmitting the infection to other cows within the herd.

Typically, 10–12% of clinical mastitis cases are attributed to *S. aureus* [44,45]. The pathogen exhibits a limited response to in vivo treatment, and *S. aureus* typically remains present in the udder. The most recognized form of resistance pertains to penicillin antibiotics; however, the level of resistance fluctuates across different study years and countries [38].

Mastitis induced by *S. aureus* is typically subclinical and chronic, manifesting more frequently during late breastfeeding than early lactation. Infection by *S. aureus* significantly affected milk supply more adversely during the first or second lactation compared to the third and later lactations [42]. The most substantial milk losses transpired during the first and third lactations, while the effects were diminished after the second lactation [46].

Antimicrobials commonly utilized in veterinary medicine continue to be legally acquired in Romania without the necessity of a veterinary prescription. This may contribute to the inappropriate and excessive application of antimicrobials in dairy herds, potentially leading to persistent infections and the development of antimicrobial resistance [29,47]. This significant issue concerning animal and public health can lead to direct financial losses for dairy farmers, stemming from medication expenses and milk wastage during treatment. The thorough and precise identification of microorganisms in buffalo mastitis is crucial for determining the reservoirs and sources of infection, as well as for implementing suitable hygienic measures [48,49]. Consequently, effective management practices, along with the judicious use of antimicrobials, are essential to prevent the development of bacterial resistance in human dairy products.

Pascu et al. [33] reported in a study on the etiology of mastitis and antimicrobial resistance in dairy cattle farms in Western Romania that they found *Staphylococcus* spp. (43.19%) to be the most prevalent isolated bacteria, followed by *Streptococcus* spp. (22.41%), *E. coli* (13.79%), *Corynebacterium* spp. (7.75%), *Enterococcus* spp. (8.62%), and *Enterobacter* spp. (4.31%). Antimicrobial resistance profiling indicated low susceptibility of Gram-positive bacteria to most tested antimicrobials, except cephalothin. Most isolates were multidrug-resistant (MDR), with resistance patterns that included commonly used antimicrobials for treating cow mastitis in Romania.

In another study, Iancu et al. [37] investigated the prevalence and etiology of subclinical mastitis in Țurcana sheep flocks located in Southwestern Romania. Thus, *Staphylococcus aureus* (129/146; 88.4%) was identified as the predominant pathogen, followed by *Streptococcus* spp. (14/146; 9.6%), *Enterococcus* spp. (7/146; 4.8%), *Pseudomonas aeruginosa* (6/146; 4.1%), and *Klebsiella pneumoniae* (5/146; 3.4%).

Also, in the western part of Romania, Degi et al. [24] conducted a preliminary study on the antibacterial profile of staphylococcal isolates associated with mastitis in buffaloes. Between January and May 2017, 68 samples of milk were collected from bubalines reared for milk production in the southwestern part of Arad County, Pecica. Following the evaluation of the milk samples, 26 strains of *Staphylococcus* were isolated, comprising 19 coagulase-positive (*S. hyicus* and *S. aureus*) and 7 coagulase-negative strains (*S. haemolyticus*, *S. sciuri*, and *S. epidermidis*). The antibiotic susceptibility of these *Staphylococcus* strains isolated from mastitic milk was variable, depending on the antibiotic group. For the β-lactams used (methicillin, ceftriaxone, cefoxitin, cefaclor, and ampicillin with sulbactam), the antibiotic sensitivity was maximal, except for methicillin, where resistant strains were isolated. All the isolated strains were resistant to polymyxin B and sensitive to ciprofloxacin.

However, the findings presented here are crucial in demonstrating the primary *S. aureus* linked to Romanian buffalo mastitis in a significant milk-producing area of Western Romania. Further studies are needed to validate these results across dairy farms in various regions to understand mastitis in Romania better.

Compared to cows, studies highlighting buffalo milking significantly emphasize the need to enhance monitoring of mastitis (e.g., *Staphylococcal* mastitis) development and environmental factors. Therefore, implementing effective milking practices, understanding potential sources of pathogenic microorganisms associated with harmful zoonoses, and using personal protective equipment represent the best strategies in buffalo milking parlors. This goal can be achieved through ongoing and thorough information and training for livestock operators. Gaining a deeper understanding of the challenges related to the Romanian buffalo breeding system and the measures that inform about potential biological risks for employees is the most effective approach to achieving tangible results for worker safety.

This study has several limitations. First is the small convenience sample. Few Romanian buffalo farms exist, thus creating an inherent challenge to studying zoonotic transmission in buffalo production facilities. Additionally, this study focused on detecting *S. aureus* among clinical mastitis in Romanian buffalo in Western Romania. This study did not investigate the epidemiological transmission of MRSA in Romania. Future studies should include sampling buffalo milk on more farms and with farmers and workers, and buffalo milk dairy production areas should also be considered for further investigation.

## 4. Materials and Methods

### 4.1. Romanian Buffalo Farms and Sample Collection

Romanian buffalo with a history of clinical mastitis were chosen for this study. Clinical mastitis is defined as mastitis detectable by visible changes in the milk, such as abnormal color or clots that persist for longer than three hand strips; changes to the quarter, including heat, redness, or swelling; changes in the buffalo, such as lethargy or systemic illness; or any combination of these indicators. Individual buffalo were eligible to be sampled multiple times throughout the study. These animals came from farms in a significant dairy region of Western Romania, specifically the Banat region. Veterinarians collected milk samples between April 2023 and September 2024. Selective teats that displayed visible milk alterations or yielded a positive result in the California Mastitis Test Kit (CMT, BioCore Diagnostics GmbH, Germany) were washed, dried with a paper towel, and disinfected with 70% alcohol [50]. Three streams of milk were collected in sterile tubes (Milk Sample Vial, 35 mL, Movora, Zürich, Switzerland), immediately frozen, and sent to the diagnostic laboratory (Laboratory of Bacterial Diseases) at the Faculty of Veterinary Medicine in Timișoara.

**Figure 1:**
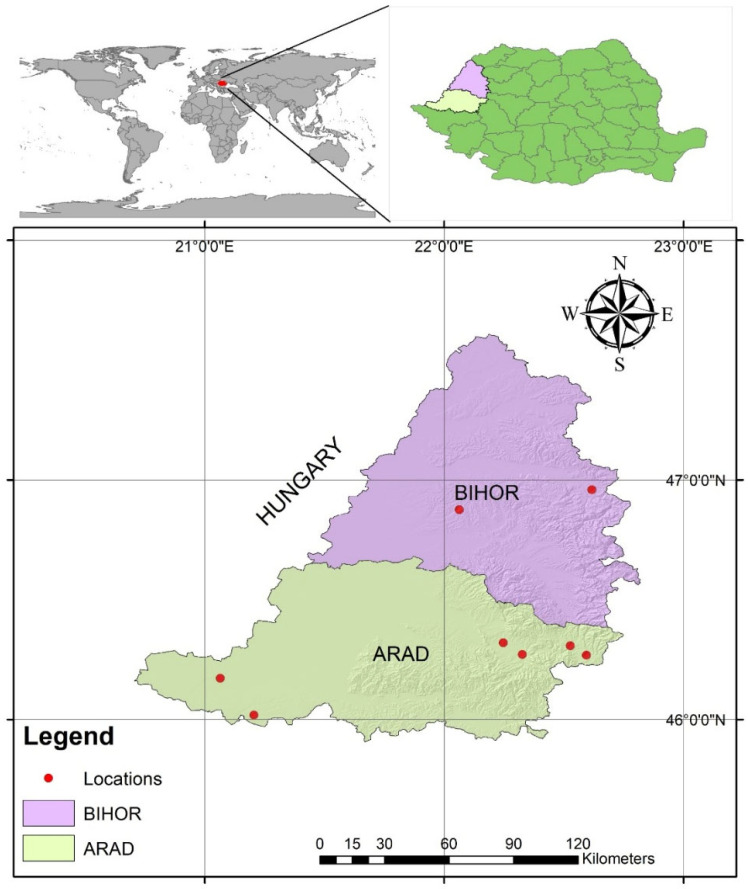
The geographical distribution of farms included in the study.

Table 4 provides details on the number of buffaloes analyzed per farm, while Figure 1 illustrates the geographical distribution of the buffalo farms included in the study.

The locations where the samples were collected included six farms and one microfarm (established as a breeders’ association, consisting of several households) in Arad and Bihor counties (Pecica, Arad; Lăzăreni, Bihor; Vinga, Arad; Beznea (Bratca), Bihor; Gurahonț, Arad; Hălmagiu, Arad; Vârfurile, Arad;and Rădești (Dieci), Arad) and one breeders association microfarm with a total animal population of 254 animals and 75 milk samples analyzed. Each farm had an average of 31.75 (±23.41) animals, and the average number of milk samples collected per farm was 9.38 (±14.01). The highest average milk sampling was in Beznea, Bihor, with 0.57 samples per animal, while the minimum was in Rădești (Dieci), Arad, with only 0.086 samples per animal. The differences in sampling may indicate different approaches to herd management, milk production, or health monitoring. In this study, two milk samples were collected from the same buffalo in cases where a customized quarter-based treatment was administered based on the anamnesis findings. The samples were collected from a single farm on a single occasion, rather than multiple times. Furthermore, sampling was typically conducted during the animal’s regular milking time to minimize discomfort and ensure compliance with standard management practices.

### 4.2. Bacteriological Culture

This study involved the cultivation of bacteria from mastitic milk samples of Romanian buffalo, conducted as a standard diagnostic service for buffalo farms in Western Romania. From April 2023 to September 2024, this study encompassed six farms situated in diverse areas of Western Romania. The year-round farms maintained a herd size ranging from 30 to 75 buffalo (Romanian buffalo), with 17 to 43 animals being milking buffalo. The operations were equipped with milking equipment from DeLaval, Sweden. During this period, 115 milk samples from 75 Romanian buffaloes of different ages were collected for bacteriological examinations.

The bacterial culture was conducted using protocols established by the National Mastitis Council [49]. Ten microliters of each milk sample were plated on Columbia agar with 5% sheep blood and MacConkey agar (Oxoid S.p.A., Milan, Italy). We kept the cultures at 37 °C for a duration of 24–48 h under aerobic conditions. The fluid samples were considered positive upon the observation of one or more colonies (≥100 CFU/mL) [44]. Upon identifying three or more distinct colony types on the agar plate, contamination was presumed, leading to the dismissal of the results [43].

Conventional bacteriological methods initially conducted presumptive identification of bacterial species based on previously outlined criteria [51]. The identification of colonies was based on the physical characteristics of the colonies and through the process of Gram staining. The evaluation of hemolysis and catalase activity was conducted on Gram-positive cocci, specifically those presumed to be *Staphylococcus* spp. The isolates underwent analysis for coagulase activity. In our study, we utilized the Coagulase Test (Tubes; Merck KGaA, Darmstadt, Germany) and the Staphylase^TM^ Test (Oxoid, Thermo Fisher Scientific Inc., Bucharest, Romania), respectively, to differentiate potentially pathogenic *Staphylococcus* species from other Gram-positive, catalase-positive cocci, and for the rapid and accurate identification of *S. aureus*, based on the detection of coagulase and clumping factor. This test enables the differentiation of pathogenic species from commensal ones, playing a crucial role in diagnosing infections and the epidemiological analysis of isolated strains.

We performed the urease test using the commercial kit (Infectious disease test strip Ut0050, TestLine Clinical Diagnostics, Czech Republic) to differentiate *S. shleiferi* from *S. coagulans*. This biochemical assay was performed to ensure accurate species identification, as *S. coagulans* is known to exhibit urease activity, whereas *S. schleiferi* typically does not.

Ultimately, the species of the isolates were confirmed using Vitek 2 Compact systems (Bio Mérieux, Marcy-l’Étoile, France). The subcultures obtained were analyzed to identify the staphylococcal species utilizing the GP ID cards from the Vitek^®^ 2 automatic system (identification panel, GP ID card; software, Vitek^®^ 2 Systems version 07.01; bioMérieux, Marcy l’Etoile, France) following the manufacturer’s standard protocols.

The automated Vitek 2^®^ Compact system (bioMérieux, Marcy l’Etoile, France) was employed to analyze a comprehensive antimicrobial susceptibility profile by determining the minimum inhibitory concentration (MIC) using veterinary susceptibility AST-GP79 card Livestock WW panels (BioMériux, Inc., Durham, NC, USA) for Gram-positive bacteria in conjunction with the Vitek^®^ 2 Advanced Expert System software (version 8.01). The Vitek 2^®^ kit evaluated a total of 18 antimicrobial substances (minimum inhibitory concentrations (MICs] from 7 classes were included in the study accordingly: ß lactams—benzylpenicillin (PCG; 0.03–0.5 µg/mL), oxacillin (OXA; 0.25–4 µg/mL), ampicillin (AM; 0.25–32 µg/mL), cefalotin (CF; 2–32 µg/mL), ceftiofur (CFT; 0.5–8 µg/mL), and cefquinome (CFQ; 1–64 µg/mL); aminoglycosides—gentamicin (GM; 0.5–16 µg/mL), amikacin (AN; 2–64 µg/mL), kanamycin (K; 4–64 µg/mL), and neomycin (N; 2–32 µg/mL); quinolones—enrofloxacin (ENR; 0.5–4 µg/mL); tetracyclines—tetracycline (TE; 1–16 µg/mL); macrolides—erythromycin (E; 0.25–8 µg/mL), tilmicosin (TIL; 0.25–4 µg/mL), and tylosin (TI; 1–32 µg/mL); lincomycins—clindamycin (CLI; 0.125–4 µg/mL); sulfonamides—trimethoprim/sulfamethoxazole (SXT; 10 (0.5/9.5)–320 (16/304) µg/mL); and amphenicols—florfenicol (FFC; 4–32 µg/mL). Only the MIC breakpoint for susceptibility was considered in the study, according to the CLSI M100 (or CLSI VET01-A5, depending on context) guidelines (Tables S1 and S2).

The card also includes three additional tests: the cefoxitin screen test, inducible clindamycin resistance, and the high-level synergism streptomycin test. Our study identified 19 *Staphylococcus* isolates, 8 *S. aureus* and 11 *S. hyicus*, as methicillin-resistant based on the oxacillin screening test (OXSF). The prevalence of methicillin-resistant *Staphylococcus* strains was 22.89% (19/83), with a 95% confidence interval ranging from 13.85% to 31.93%, and a standard deviation of 3.83.

*Staphylococcus* spp. isolates were classified as multidrug-resistant (MDR) when they exhibited resistance to at least one antimicrobial agent in three or more different antimicrobial classes. Moreover, the resistance of *S. aureus* to penicillin was omitted from the criteria for multidrug resistance due to the prevalent resistance of *S. aureus* to this antimicrobial agent [29,52].

An in-depth analysis of antimicrobial multidrug-resistant phenotypes was performed on the identified *Staphylococcus* pathogens, specifically *S. aureus*, *S. hyicus*, and *S. schleiferi*. MDR refers to the development of resistance to at least one antimicrobial agent within three or more classes of antimicrobials.

All acquired data were statistically analyzed.

### 4.3. Analysis of Statistics

The information gathered was statistically examined using Microsoft Office Excel (Microsoft Corporation, Redmond, WA, USA). The results provided refer to the 95% confidence interval and *p*-value, as obtained from the Chi-Squared test and standard deviation (mean, respectively).

## 5. Conclusions

Mastitis poses an increasing threat to the dairy industry and is linked to significant economic losses. The capacity of mastitis causing *S. aureus* to develop resistance to commonly used antimicrobials, its ability to form biofilms, and its ability to invade and survive within mammary epithelial cells further complicate the issue and render the antibiotics used for mastitis ineffective. Every *Staphylococcus* species should be considered unique in its capacity to cause mastitis, requiring the development of suitable control strategies based on species-specific knowledge. The current approaches to treating *Staphylococcus* spp.-caused mastitis are not successful. Developing efficient and long-lasting non-antibiotic control methods, such as vaccines, preventative treatments, and other novel strategies, requires a deeper comprehension of the virulence factors, pathophysiology, and host immunological responses in Romanian buffalo-adapted strains of *Staphylococcus* spp., which should constitute approaches for future research in this field.

## Figures and Tables

**Table 1:** Distribution frequency of *Staphylococcus* spp. as determined by Vitek^®^ 2 system identification (Vitek 2^®^ GP ID card).

*Staphylococcus* spp.	No. of Isolated Species (*n* = 83)	% (95% CI)	*p*-Value/Chi-Squared
*S. aureus*	46	55.42 (44.72–66.12%)	0.002/12.14
*S. hyicus*	28	33.73 (23.54–43.92%)	0.95/0.004
*S. schleiferi*	9	10.84 (4.06–17.62%)	0.002/12.61

**Table 2:** Results of the antimicrobial susceptibility testing for *Staphylococcus* spp. Identified from clinical mastitis, as determined by Vitek^®^ 2 (AST-GP79), Gram-positive Livestock WW (Ref 421825, BioMérieux, France).

Antibiotics (Drugs Family)	*S. aureus* (*n* = 46)		*S. hyicus* (*n* = 28)		*S. schleiferi* (*n* = 9)	
	S	R	S	R	S	R
AM (BL)	60.86 *	39.13	39.28	60.71	66.66	33.34
	(28)	(18)	(11)	(17)	(6)	(3)
P (BL)	36.95	63.04	39.28	60.71	22.23	77.77
	(17)	(29)	(11)	(17)	(2)	(7)
CF (BL)	73.91	26.08	53.57	46.42	77.77	22.23
	(34)	(12)	(15)	(13)	(7)	(2)
CFT (BL)	89.13	10.86	53.57	50.0	55.55	44.45
	(41)	(5)	(15)	(14)	(5)	(4)
OX1 (BL)	82.60	17.39	60.71	39.28	100	(0)
	(38)	(8)	(17)	(11)	(9)	
CEQ (BL)	95.65	4.34	100	0	100	(0)
	(44)	(2)	(28)	(0)	(9)	
OXSF	8 Positive		11 Positive		Negative	
GM (AM)	41.30	58.69	32.14	67.85	33.34	66.66
	(19)	(27)	(9)	(19)	(3)	(6)
AN (AM)	54.34	45.65	46.42	53.57	33.34	66.66
	(25)	(21)	(13)	(15)	(4)	(5)
K (AM)	50.0	50.0	35.71	64.28	44.45	55.55
	(23)	(23)	(10)	(18)	(4)	(5)
N (AM)	28.26	71.73	25.0	75.0	22.23	77.77
	(13)	(33)	(7)	(21)	(2)	(7)
HLS (AM)	76.08	23.91	39.28	60.71	44.45	55.55
	(35)	(11)	(11)	(17)	(4)	(5)
ENR (FQ)	80.43	19.56	100	0	88.88	11.12
	(37)	(9)	(28)	(0)	(8)	(1)
TE (TE)	19.56	80.43	14.28	85.71	22.23	77.77
	(9)	(37)	(4)	(24)	(2)	(7)
E (MA)	39.13	60.86	28.57	71.42	33.34	66.66
	(18)	(28)	(8)	(20)	(3)	(6)
TIL (MA)	45.65	54.34	39.28	60.71	44.45	55.55
	(21)	(25)	(11)	(17)	(4)	(5)
TI (MA)	56.52	47.82	64.28	35.71	44.45	55.55
	(26)	(22)	(18)	(10)	(4)	(5)
ICR	Negative		Negative		Negative	
C (Li)	67.39	32.60	67.85	32.14	77.77	22.23
	(31)	(15)	(19)	(9)	(7)	(2)
SXT (TS)	26.08	73.91	25.0	75.0	22.23	77.77
	(12)	(34)	(7)	(21)	(2)	(7)
FFC (AMF)	63.04	36.95	53.57	46.42	55.55	44.45
	(29)	(17)	(15)	(13)	(5)	(4)

* The exact number and percentage of bacterial strains are expressed. Abbreviations for antimicrobials: AM—ampicillin; P—benzylpenicillin G; CF—cefalotin; CFT—ceftiofur; CFQ—cefquinome; OX1—oxacillin; OXSF—oxacillin screening test; GM—gentamicin; AN-amikacin; K—kanamycin; N—neomycin; ENR—enrofloxacin; TE—tetracycline; E—erythromycin; ICR—inducible clindamycin resistance; FFC—florfenicol; C—clindamycin; SXT—sulfamethoxazole-trimethoprim; TIL—tilmicosin; TI—tylosin; HLS—high-level synergism streptomycin. S: susceptible; R: resistant. Pharmaceutical abbreviations for family terms: BL—β-lactams; AM—aminoglycosides; FQ—fluoroquinolones; TE—tetracyclines; MA—macrolides; Li—lincosamides; TS—sulfonamides; AMF—phenicols.

**Table 3:** Patterns of resistance in *Staphylococcus* spp. (*n* = 83), obtained from buffalo mastitis conditions, exhibiting resistance to at least three antimicrobial classes.

No.	*Staphylococcus* spp.	No. of Isolates	Resistance to Antimicrobial Profile	No. of Classes of Antimicrobials
1.	*Staphylococcus aureus*	2	AN, AM, P, CF, CFT, CFQ, OX1, GM, K, N, ENR, TE, E, FFC, C, SXT, TIL, TI	8
2.	*Staphylococcus aureus*	3	AN, AM, P, CF, CFT, OX1, GM, K, N, ENR, TE, E, FFC, C, SXT, TIL, TI	8
3.	*Staphylococcus aureus*	3	AN, AM, P, CF, OX1, GM, K, N, ENR, TE, E, FFC, C, SXT, TIL, TI	8
4.	*Staphylococcus aureus*	1	AN, AM, P, CF, GM, K, N, ENR, TE, E, FFC, C, SXT, TIL, TI	8
5.	*Staphylococcus aureus*	3	AN, AM, P, CF, GM, K, N, TE, E, FFC, C, SXT, TIL, TI	7
6.	*Staphylococcus aureus*	3	AN, AM, P, GM, K, N, TE, E, FFC, C, SXT, TIL, TI	7
7.	*Staphylococcus aureus*	2	AN, AM, P, GM, K, N, TE, E, FFC, SXT, TIL, TI	6
8.	*Staphylococcus aureus*	1	AN, AM, P, GM, K, N, TE, E, SXT, TIL, TI	5
9.	*Staphylococcus aureus*	3	AN, P, GM, K, N, TE, E, SXT, TIL, TI	5
10.	*Staphylococcus aureus*	1	P, GM, K, N, TE, E, SXT, TIL, TI	5
11.	*Staphylococcus aureus*	1	P, GM, K, N, TE, E, SXT, TI	5
12.	*Staphylococcus aureus*	2	P, GM, N, TE, E, SXT, TI	5
13.	*Staphylococcus aureus*	2	P, GM, N, TE, E, SXT	5
14.	*Staphylococcus aureus*	1	P, N, TE, E, SXT	5
15.	*Staphylococcus aureus*	1	P, N, TE, SXT	4
16.	*Staphylococcus aureus*	4	N, TE, SXT	3
17.	*Staphylococcus hyicus*	9	AN, AM, P, CF, CFT, OX1, GM, K, N, TE, E, FFC, C, SXT, TIL, TI	7
18.	*Staphylococcus hyicus*	1	AN, AM, P, CF, CFT, OX1, GM, K, N, TE, E, FFC, SXT, TIL, TI	6
19.	*Staphylococcus hyicus*	1	AN, AM, P, CF, CFT, OX1, GM, K, N, TE, E, FFC, SXT, TIL	6
20.	*Staphylococcus hyicus*	2	AN, AM, P, CF, CFT, GM, K, N, TE, E, FFC, SXT, TIL	6
21.	*Staphylococcus hyicus*	1	AN, AM, P, CFT, GM, K, N, TE, E, SXT, TIL	5
22.	*Staphylococcus hyicus*	1	AN, AM, P, GM, K, N, TE, E, SXT, TIL	5
23.	*Staphylococcus hyicus*	2	AM, P, GM, K, N, TE, E, SXT, TIL	5
24.	*Staphylococcus hyicus*	1	GM, K, N, TE, E, SXT,	4
25.	*Staphylococcus hyicus*	1	GM, N, TE, E, SXT,	4
26.	*Staphylococcus hyicus*	1	N, TE, E, SXT,	4
27.	*Staphylococcus hyicus*	1	N, TE, SXT,	3
28.	*Staphylococcus schleiferi*	1	AN, AM, P, CF, CFT, GM, K, N, ENR, TE, E, FFC, C, SXT, TIL, TI	8
29.	*Staphylococcus schleiferi*	1	AN, AM, P, CF, CFT, GM, K, N, TE, E, FFC, C, SXT, TIL, TI	7
30.	*Staphylococcus schleiferi*	1	AN, AM, P, CFT, GM, K, N, TE, E, FFC, SXT, TIL, TI	6
31.	*Staphylococcus schleiferi*	1	AN, P, CFT, GM, K, N, TE, E, FFC, SXT, TIL, TI	6
32.	*Staphylococcus schleiferi*	1	AN, P, CFT, GM, K, N, TE, E, SXT, TIL, TI	5
33.	*Staphylococcus schleiferi*	1	P, CFT, GM, N, TE, E, SXT, TIL, TI	5
34.	*Staphylococcus schleiferi*	1	P, CFT, N, TE, SXT, TIL, TI	5

Legend: AN—amikacin; AM—ampicillin; P—benzylpenicillin G; CF—cefalotin; CFTceftiofur; CFQcefquinome; OX1—oxacillin; GM—gentamicin; Kkanamycin; Nneomycin; ENRenrofloxacin; TE—tetracycline; E—erythromycin; FFC—florfenicol; C—clindamycin; SXT—sulfamethoxazole-trimethoprim; TIL—tilmicosin and TI—tylosin.

**Table 4:** Herd size and milk sample collection ratio in farms and microfarms.

No.	Farm/Microfarm Location	Livestock	Number of Milk Samples Collected	Milk Samples per Animal Ratio
1.	Pecica-Arad	56	8	0.142857
2.	Lăzăreni-Bihor	30	5	0.166667
3.	Vinga-Arad	25	11	0.440000
4.	Beznea (Bratca)-Bihor	75	43	0.573333
5. Microfarm, breeders’ association	Gurahonț-Arad	10	2	0.200000
	Hălmagiu-Arad	8	1	0.125000
	Vărfurile-Arad	15	2	0.133333
6.	Rădești (Dieci)-Arad	35	3	0.085714
Total		254	75	

## Data Availability

The data supporting this study’s findings are available from the corresponding author upon reasonable request.

## Supplementary Materials

The following supporting information can be downloaded at: https://www.mdpi.com/article/10.3390/antibiotics14060537/s1, Table S1 (Breakpoints for *Staphylococcus* spp.—CLSI VET01). Table S2 (Breakpoints—CLSI M100-S24—for *Staphylococcus* spp. and other relevant pathogens).
