# Supplementary material for: Antimicrobial Susceptibility Patterns of Staphylococcus spp. Isolates from Mastitic Cases in Romanian Buffaloes from Western Romania
Source: Antibiotics (Basel). 2025 May 23;14(6):537. doi: 10.3390/antibiotics14060537 (PMC12189436; doi:10.3390/antibiotics14060537)
Supplement: Supplementary file 1 [file antibiotics-14-00537-s001.zip › antibiotics-3531263-supplementary.pdf]

The clinical breakpoint values for cefquinome, florfenicol, tilmicosin, tylosin, kanamycin, and neomycin agents, in the case of *Staphylococcus* spp. isolated from animals, are provided in the CLSI VET01 guidelines (Clinical and Laboratory Standards Institute – Performance Standards for Antimicrobial Disk and Dilution Susceptibility Tests for Bacteria Isolated from Animals). Below are the relevant values according to the latest available version (CLSI VET01, edition 5 or VET01S, updated edition):

These agents are predominantly used in veterinary medicine, while EUCAST mainly focuses on establishing breakpoints for using antimicrobials in human medicine. For antimicrobials specific to the veterinary field, such as those mentioned, breakpoints are often provided by organizations such as CLSI (Clinical and Laboratory Standards Institute) in dedicated documents, such as CLSI VET01.

Table S1. Breakpoints for *Staphylococcus* spp. – CLSI VET01

**Antibiotic**	**Animal Species (Target)**	**Testing Method**	**Breakpoint (μg/mL or mm)**	**Comments**
Cefquinome	Bovine (mastitis)	MIC	≤ 2 μg/mL	Only for bovine mastitis caused by *Staphylococcus aureus*
Florfenicol	Porcine	MIC	≤ 2 μg/mL	Only for respiratory pathogens; no data for *Staphylococcus* spp.
Tilmicosin	Porcine	MIC	≤ 16 μg/mL	Not validated for *Staphylococcus* spp.; only for respiratory pathogens
Tylosin	-	-	Not available	No established breakpoints for *Staphylococcus* spp.
Kanamycin	-	-	Not available	No official breakpoints for *Staphylococcus* spp.
Neomycin	-	-	Not available	No official breakpoints for *Staphylococcus* spp.

This table summarizes the current Clinical Breakpoints for selected antimicrobial agents against *Staphylococcus* spp. based on the CLSI VET01 guidelines. These values primarily apply to veterinary pathogens and must be interpreted within the specific context of the target animal species and infection site.

Table S2: Breakpoints – CLSI M100-S24 – for *Staphylococcus* spp. and other relevant pathogens

**Class**	**Antibiotic**	**Target Species**	**S ≤ (μg/mL or mm)**	**R ≥ (μg/mL or mm)**	**Remarks**
Beta-lactams	Ampicillin (AM)	*Staphylococcus* spp.	≤ 0.25	≥ 0.5	

		Enterococcus spp.	≤ 8	≥ 16	
	Benzylpenicillin G (P)	*Staphylococcus* spp.	≤ 0.12	≥ 0.25	
		Streptococcus spp.	≤ 0.12	≥ 0.25	
	Cephalothin (CF)	*Staphylococcus* spp.	≤ 8	≥ 32	
	Ceftiofur (CFT)	*Staphylococcus* spp.	≤ 2	≥ 8	
	Cefquinome (CFQ)	-	-	-	Breakpoint values not defined
	Oxacillin (OX1)	*Staphylococcus aureus*	≤ 2	≥ 4	
	Cefoxitin (OXSF)	*Staphylococcus aureus*	≥ 22 mm	≤ 21 mm	Disc diffusion test
Aminoglycosides	Gentamicin (GM)	*Staphylococcus* spp.	≤ 4	≥ 16	
	Amikacin (AN)	*Staphylococcus* spp.	≤ 16	≥ 64	
	Kanamycin (K)	-	-	-	Breakpoint values not defined
	Neomycin (N)	-	-	-	Breakpoint values not defined
Other classes	Enrofloxacin (ENR)	*Staphylococcus* spp.	≤ 0.5	≥ 2	
	Tetracycline (TE)	*Staphylococcus* spp.	≤ 4	≥ 16	
	Erythromycin (E)	*Staphylococcus* spp.	≤ 0.5	≥ 8	
	Clindamycin (C)	*Staphylococcus* spp.	≤ 0.5	≥ 4	
	Florfenicol (FFC)	-	-	-	Breakpoint values not defined
	Sulfamethoxazole-trimethoprim (SXT)	*Staphylococcus* spp.	≤ 2/38	≥ 4/76	
	Tilmicosin (TIL)	-	-	-	Breakpoint values not defined
	Tylosin (TI)	-	-	-	Breakpoint values not defined
	Streptomycin (HLS)	-	-	-	High-level synergy

Table S2 below summarizes the breakpoint values for several antibiotic classes, based on CLSI M100-S24 guidelines. These refer primarily to *Staphylococcus* spp. and additional pathogens commonly encountered in clinical microbiology.

Reference:

27. Clinical and Laboratory Standards Institute (CLSI). (2024). Performance Standards for Antimicrobial Susceptibility Testing. CLSI Supplement M100-S24. Wayne, PA: CLSI.
